# Molecular survey and phylogenetic analysis of *Babesia vogeli* in dogs

**DOI:** 10.1038/s41598-022-11079-x

**Published:** 2022-04-28

**Authors:** Abdelfattah Selim, Ameer Megahed, Mourad Ben Said, Abdullah D. Alanazi, Mohamed Z. Sayed-Ahmed

**Affiliations:** 1 Department of Animal Medicine (Infectious Diseases), Faculty of Veterinary Medicine, Benha University, Toukh, 13736 Egypt; 2 Department of Animal Medicine (Internal Medicine), Faculty of Veterinary Medicine, Benha University, Moshtohor-Toukh, Kalyobiya, 13736 Egypt; 3 Department of Veterinary Clinical Medicine, College of Veterinary Medicine, University of Illinois at Urbana-Champaign, Champaign, Illinois 61802 USA; 4 Higher Institute of Biotechnology of Sidi Thabet, University of Manouba, 2010 Manouba, Tunisia; 5 Laboratory of Microbiology at the National School of Veterinary Medicine of Sidi Thabet, University of Manouba, 2010 Manouba, Tunisia; 6 Department of Biological Science, Faculty of Science and Humanities, Shaqra Univerisity, 1040, Ad-Dawadimi, 11911 Saudi Arabia; 7 Department of Pharmacy Practice, College of Pharmacy, Jazan University, Jizan, 82722 Saudi Arabia; 8 Department of Internal Medicine and Infectious Diseases, Faculty of Veterinary Medicine, Mansoura University, Mansoura, 35516 Egypt

**Keywords:** Molecular biology, Risk factors

## Abstract

Canine babesiosis is a life-threatening haemoparasitic disease in dogs that is prevalent worldwide. In this study, the prevalence of *Babesia vogeli* (*B. vogeli*) was investigated in dogs from Egypt by using Polymerase Chain Reaction (PCR) assay, and associated risk factors were evaluated. In addition, phylogenetic position of *B. vogeli* Egyptian isolate was determined by sequencing. A total of 275 blood samples were taken from dogs located in four governorates belonging to the north of Egypt. Samples were examined by PCR targeting the *B. vogeli* 18S rRNA gene and this species was also confirmed by sequencing. Overall, the prevalence of *B. vogeli* was 5.1% among the studied dogs and the highest prevalence rate was found in the Giza governorate. Univariate logistic regression was used to evaluate each variable individually. The results revealed a significant association between the prevalence of *B. vogeli* infection and whether or not dogs were infested with ticks and the type of floor used in dog shelters. Additionally, tick infestation (OR 6.1, 95% CI 1.2–31.4), and living in shelters with soil floors (OR 3.8, 95% CI 0.8–17.8) were identified as potential risk factors for *B. vogeli* infection. Phylogenetic analysis was performed using *B. vogeli* 18S rRNA partial sequences with the hypervariable V4 region from GenBank. The Egyptian isolate was assigned to second sub-cluster with *B. vogeli* isolates from Japan, Venezuela and Paraguay within the *B. vogeli*/*B. canis* cluster. The present data will be useful to improve the understanding of canine babesiosis epidemiology and ways to control the disease in companion dogs.

## Introduction

Babesiosis is a disease that affects dogs all over the world. It is caused by intracellular erythrocytic parasites of the genus *Babesia*^1,2^. The disease is spread by *ixodid* ticks that carry either small (1–2.5 µm) or large (4-5 µm) *Babesia* species. Large *Babesia* spp. were previously thought to be *B. canis*, but they are now classified into three independent species based on their genetic traits, the severity of the clinical signs that they cause, their tick vectors and their range geographical distribution^3–5^. These three species are *Babesia canis, Babesia rossi* and *Babesia vogeli*^2,6,7^.

The most common *Babesia* species in Europe is *B. canis*, which is transmitted by *Dermacentor reticulatus. Haemaphysalis elliptica* transmits *B. rossi,* which is the most common species in South Africa; *B. vogeli* is transmitted by *Rhipicephalus sanguineus* sensu lato, which is most commonly found in tropical and subtropical areas^1,8–10^.

Canine babesiosis has no specific symptoms; however, pyrexia, anorexia, epistaxis, petechiae and splenomegaly are common signs, along with hemoglobinuria, anemia and thrombocytopenia^2^. The disease is routinely diagnosed based on clinical signs, hematological findings and detection of intracellular parasites in blood smears^11,12^.

In order to confirm infection and start treatment, various laboratory procedures, such as serological tests and molecular methods, should be performed due to the lack of distinct clinical signs and the high frequency of false negatives that occur in analysis of blood smears, especially in cases of low parasitaemia^13,14^. Serological testing is a valuable diagnostic tool, although it has limits due to cross-reactivity between *Babesia* species and the inability to use this method to differentiate early from chronic infection^15^.

To directly detect hemoparasite DNA from clinical or environmental samples, a variety of molecular tools, such as loop-mediated isothermal amplification assay (LAMP), quantitative polymerase chain reaction (qPCR), and high resolution melting analysis (HRM), are used, providing relatively inexpensive, rapid molecular tests with high throughput. The PCR assay is more reliable than traditional methods for detection of piroplasms and has high sensitivity and specificity^16–21^.

Sparse research has been done on canine babesiosis or its associated risk factors in Egypt. A few studies have detected *B. vogeli* in dogs from Egypt based on microscopic smear and molecular techniques^22,23^. However, these researchers did not consider the risk factors associated with *B. vogeli* infection in dogs.

Therefore, the study described here aimed to determine the prevalence of canine babesiosis and its associated risk factors based on PCR assays and partial sequencing of the 18S rRNA gene.

## Materials and methods

### Ethical statement

All procedures involving the handling and collection of blood samples were approved by the Benha University ethical committee for animal experiments. Informed consent and permissions were obtained from the dog's owners to collect samples. All procedures involving laboratory animals were performed in accordance with current standards and regulations, and approved by the ethics committee of the Faculty of Veterinary Medicine at Benha University. This study was carried out in compliance with the ARRIVE guidelines.

### Study area

The study was conducted during 2019 in the four governorates of Giza, Kafr El Sheikh, Qalyubia and Gharbia located in the north of Egypt. According to the Köppen Geiger classification, the climate of the selected areas is that of a desert characterized by hot, dry summers and mild winters. The average annual temperature is 22 °C and the annual rainfall is 180 mm during the winter season.

### Sampling

The number of samples to be taken in this study was determined based on an equation described by Thrusfield^24^ as follows:

$$n=\frac{{1.96}^{2}\times {p}_{exp}(1-{p}_{exp})}{{d}^{2}},$$

where n is the sample size, *p*_*exp*_ is the expected prevalence, which in this case was taken as 50%, and d^2^ is the precision, which was set at 5% in this study. To meet this requirement, a total of 275 blood samples were collected from saphenous and cephalic veins of dogs in sterile vacuum tubes and mixed with ethylenediamine tetraacetic acid (EDTA) buffer. All sampled dogs were owned by individuals. Some appeared to be healthy, while others showed signs of babesiosis.

To later take into account possible risk variables, information was collected regarding the locality in which each dog lived, its sex, breed, age, whether or not it was infested with ticks without determining the level of tick infestation, whether or not an acaricide had been applied and the type of floor it was sleeping on.

### Molecular analysis

For molecular diagnosis, a commercial kit (Qiagen DNeasy-tissues-blood, Valencia, CA, USA) was used to extract DNA from whole blood samples according to the manufacturer's instructions.

DNA extraction controls were used to test newly extracted samples. The extracted DNA was stored at − 20 °C until the PCR assay could be performed. All samples were examined by using the conventional PCR assay that targeted the 18SrRNA gene and employing the BAB1 BAB4 primers, as previously described by Duarte et al.^25^. The sensitivity of this PCR assay was previously evaluated and can be able to detect one *Babesia*-infected blood cell per sample.

PCR amplification was carried out in a 25 µl volume including 12.5 µl of Dream Taq green PCR master mix (2×) (Thermo Scientific, Germany), 1 µl of each primer (20 pmol/µl), 5.5 μl of ddH_2_O and 5 μl (50–150 ng) of template DNA. In addition, distilled water and (DNA positive to *B. vogeli*) were used as negative and positive controls in order to confirm PCR results. The thermal conditions were as follows: 95 °C for 10 min, followed by 35 cycles at 95 °C for 15 s, 56 °C for 30 s and 72 °C for 1 min. The PCR products were electrophoresed in a 1.5% agarose gel with ethidium bromide staining.

PCR product based on the primers RIB-19 and RIB21 of Zahler et al.^26^ for one positive sample was purified by using the QIAquick PCR Purification Kit (QIAGEN, Valencia, USA), and sequenced with the ABI PRISM BigDye TM Terminators Kit (Applied Biosystems, USA) in accordance with the manufacturer's instructions.

The Bioedit program was used to trim and edit the obtained chromatogram, and sense and antisense sequences were used to create contigs and only overlapping sequences have been selected. The obtained sequence was deposited in GenBank with accession number LC651125.

The revealed sequence was compared and aligned with 18S rRNA partial sequences for *B. vogeli* and other *Babesia* species available in GenBank by using CLUSTAL W integrated in DNAMAN software (Version 5.2.2; Lynnon Biosoft, Que., Canada). The same software was used to construct a phylogenetic tree based on the Maximum-likelihood algorithm^27^ with bootstrap analysis of 1000 iterations^28,29^.

### Statistical analysis

Univariable logistic regression was used for initial screening of investigated exposure factors associated with *Babesia* infection with *P*-value ≤ 0.20 were considered for multivariable logistic regression. Stepwise forward multivariable logistic regression was used to identify significant risk factor(s) associated with *Babesia* infection in dogs. Variable selection for stepwise forward multivariable logistic regression model was performed based on the lowest value for the Akaike information criterion (AIC). Confounding between risk factors retained in final models was examined by adding each of the variables to the model and assessing the changes in the POR (i.e., ≥ 20%) of the remaining variables in the model. Regression analysis was performed by using SAS 9.4 (SAS Inst. Inc., Cary, NC), and *P* < 0.05 was considered significant. The multicollinearity was assessed through correlation procedure, collinearity analysis (COLLIN) and Variance Inflation Factors (VIF). Application of acaricide variable was dropped to reduce collinearity with tick infestation.

## Results

In the examined group of dogs, 5.1% of animals (n = 14/275) were found positive for *B. vogeli* by PCR targeting 18S rRNA. Of these animals, 6.7% lived in Giza, 6.3% in Qalyubia, 4.1% in Kafr El Sheikh and 3.2% in Gharbia (Table 1). Univariable logistic regression results showed that host-related variables such as sex and breed of each dog were non-significantly associated with the prevalence of *B. vogeli* infection (*P* > 0.05). Indeed, *B. vogeli* infection rate was higher in males (7.6%) and mongrel breeds (9.1%) compared to females (2.3%) and the German Shepherd and Rottweiler breeds (2.1%). Moreover, the dog age and the lack of acaricides application showed no statistically significant association with the prevalence of *B. vogeli* (P > 0.05) although the highest prevalence rate observed among dogs aged > 2 to ≤ 4 years (Table 1). According to the multivariable logistic regression analysis, the tick infestation (OR 6.1, 95% CI 0.8–17.8), and the presence of an earthen floor in the dog’s shelter (OR 3.8, 95% CI 1.2–31.4) were potential risk factors for occurrence of *B. vogeli* infection (Table 2).

**Table 1:** Univariable logistic regression analysis for identification of risk factors associated with *B. vogeli* infection in 275 dogs in Egypt.

Variable	Category	N	Positive	Prevalence (%)	POR (95% CI)	*P*-value	*VIF*
Geographic location	Gharbia	62	2	3.2	1.0 (reference)	0.299	1.2
	Giza	75	5	6.7	2.1 (0.4–11.4)		
	Qalyubia	64	4	6.3	2.0 (0.4–11.3)		
	Kafr ElSheikh	74	3	4.1	1.3 (0.2–8.0)		
Gender	Female	130	3	2.3	1.0 (reference)	0.06	1.0
	Male	145	11	7.6	3.5 (0.9–12.7)		
Breed	German Shepherd	97	2	2.1	1.0 (reference)		1.1
	Rott weiler	68	2	2.1	1.4 (0.2–10.5)	0.719	
	Mongrel	110	10	9.1	4.8 (1.0–22.2)	0.048	
Age	6 to 12 months	46	1	2.2	1.0 (reference)	0.623	1.1
	> 1 to ≤ 2 years	42	1	2.4	1.1 (0.1–18.1)		
	> 2 to ≤ 4 years	109	10	9.2	4.5 (0.6–36.6)		
	> 4 years	78	2	2.6	1.2 (0.1–13.4)		
Tick infestation	No	119	2	1.7	1.0 (reference)	0.041	3.8
	Yes	156	12	7.7	4.9 (1.1–22.2)		
Application of Acaricide	Yes	132	3	2.3	1.0 (reference)	0.054	3.8
	No	143	11	7.7	3.6 (1.0–13.1)		
Shelter floor	Paved	115	2	1.7	1.0 (reference)		1.1
	Soil + paved	98	5	5.1	3.0 (0.6–16.0)	0.190	
	Soil	52	7	11.3	7.2(1.4–35.8)	0.016

**Table 2:** Multiple stepwise logistic regression analysis of potential risk factors associated with *B. vogeli* infection in dogs from Egypt.

Variable	Categories	Estimate	SE	*P*-value	POR_adj_	95% CI_OR_
Intercept		− 6.6	1.3	< 0.001	–	–
Shelter floor	Paved	Reference				
	Soil + paved	0.9	0.87	0.284	2.5	0.5–13.8
	Soil	1.8	0.84	0.031	6.1	1.2–31.4
Tick infestation	No	Reference				
	Yes	1.3	0.79	0.041	3.8	0.8–17.8

*SE* Standard error, *POR* Odds ratio, *CI* confidence interval.

**Figure 1:**
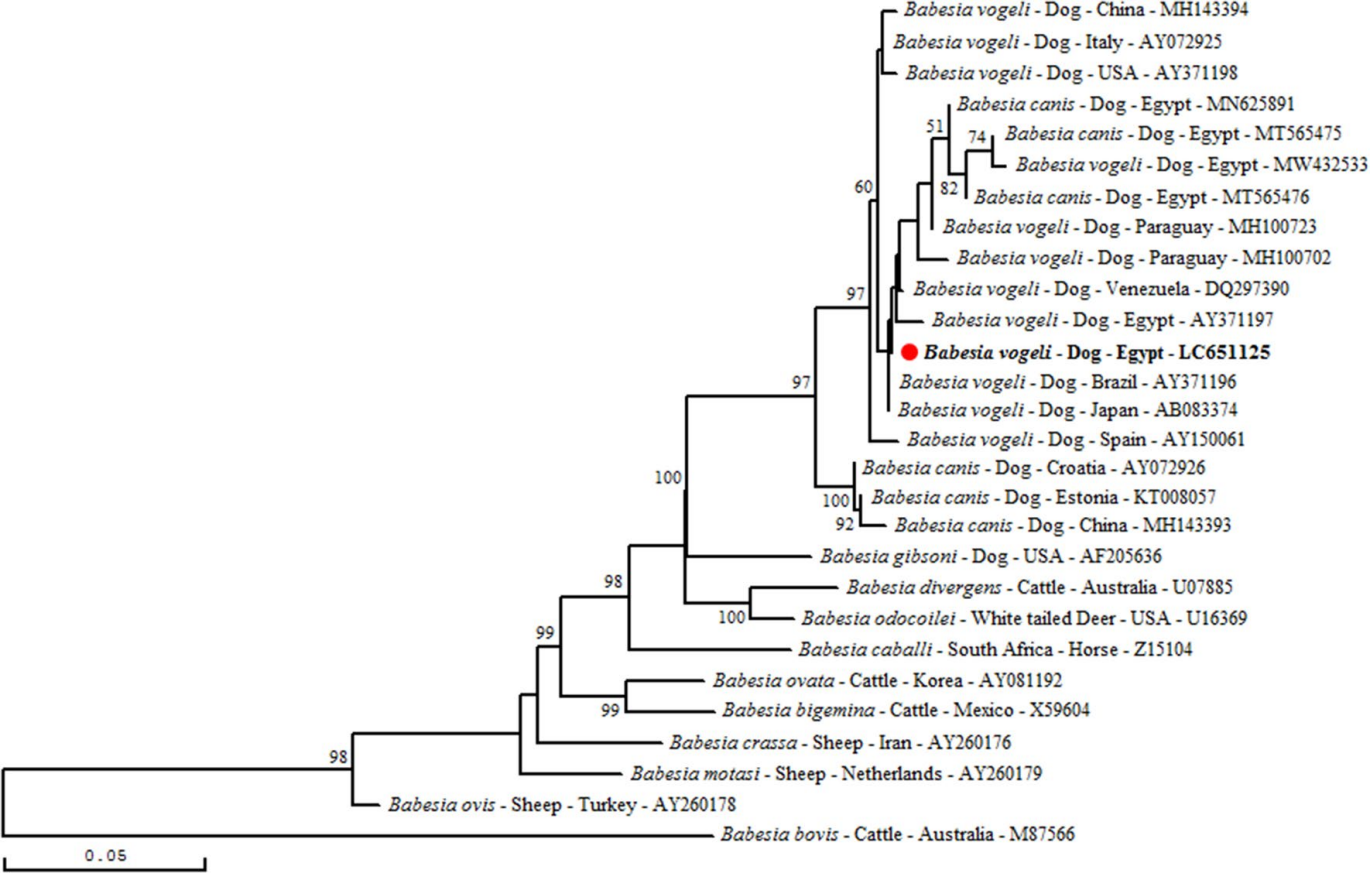
Maximum-likelihood tree based on the alignment of partial 18S rRNA sequences (531 bp) of *B. vogeli* and other sequences of *Babesia* spp. Multiple sequence alignments were generated with DNAMAN program (Version 5.2.2; Lynnon Biosoft, Que., Canada). Numbers associated with nodes represent the percentage of 1000 bootstrap iterations supporting the nodes (only percentages greater than 50% were represented). The novel sequence of *B. vogeli* obtained in the present study is represented in bold and by a circle colored in red. One *Babesia bovis* 18S rRNA partial sequence was added as out-group. The host, the country of origin and the GenBank accession number are indicated.

*Babesia vogeli* infection was confirmed by sequencing of the portion of 18SrRNA gene for one randomly selected positive dog sample. When compared our revealed sequence to those published in GenBank, the identity rates were ranged from 98 to 100%. Phylogenetic analysis was performed using *Babesia vogeli* 18S rRNA partial sequences with the hypervariable V4 region from GenBank. The Egyptian isolate was assigned to second sub-cluster with *B. vogeli* isolates from Japan, Venezuela and Paraguay within the *B. vogeli*/*B. canis* cluster closely related to that of *Babesia gibsoni* (Fig. 1).

## Discussion

*Babesia vogeli* is one of the most important pathogens among *Babesia* species found in dogs. However, few epidemiological studies have been performed on canine babesiosis in Egypt, particularly on *B. vogeli.* Many epidemiological research studies have revealed that molecular and serological approaches produce different outcomes and PCR data have been shown to be more accurate^14,30^.

The identification of DNA in many infections in vectors and hosts, particularly by haemoparasites, requires the use of molecular biology tools. The use of the PCR technique increases the ability to diagnose canine babesiosis and to provide taxonomic classification of *Babesia* species.

In dogs from four governorates in northern Egypt, the overall prevalence of *B. vogeli* infection was 5.1%. The prevalence rate varied non-significantly among localities however it was higher in Giza than other governorates. This lack of difference might be due to similarities in geo-climatic circumstances. Comparing the results with those of previous research, we found that the reported prevalence rate of *B. vogeli* was consistent with those reported in Iraq (5.1%)^31^ and in Recife, Brazil (4.8%)^32^. In contrast, the prevalence rate found in this study was lower than that estimated by Paulino et al.^33^, Khanmohammadi et al.^34^, Ćoralić et al.^1^ and Obeta et al.^35^, who reported *B. vogeli* infection rates of 15.6, 9.3, 85 and 10.8% in Brazil, Iran, Bosnia and Herzegovina, and Nigeria, respectively. Disparities between these prevalence rates could be caused by differences in experimental design, geographical or environmental factors, study duration, season of the year in which the studies were performed, sanitary measures that were applied and differences between used diagnostic tests^6,34,36–38^.

Regarding dog breeds, a higher prevalence rate of *B. vogeli* infection was recorded in mongrels (9.1%) compared to the purebreed of German Shepherd (2.1%) and Rottweiler (2.1%). This finding was in accordance with previous findings reported by Obeta et al.^35^. Interestingly, mongrels are more susceptible to tick infestation and other potential risk factors. In contrast, other research has reported that babesiosis is more common and severe in imported dogs than in native breeds. However, according to Mellanby et al.^39^, not all dog breeds are equally susceptible to babesiosis; they reported that Toy types were at lower risk than other breeds.

Nonetheless, we believe that mongrel breeds are inexpensive to purchase and their owners often ignore them and allow them to stray and scavenge, exposing them to ticks. The present findings confirm that males are more likely to be infected with *B. vogeli* than females. Similar results were found by Daniel et al.^40^, who reported that males were more susceptible to *Babesia* infection than females. The higher susceptibility of male dogs to canine babesiosis may be attributed to differences in environmental exposure to tick infestation, such as that caused by a strong tendency to roam, or sex-related genetic or hormonal effects on disease. Females were also considered to be better managed by their owners in order to get more money from their puppies^39,40^. However, other studies have shown babesiosis to be more prevalent in females than males, and researchers have attributed this to increased sitting behavior in female dogs, especially while nursing their puppies, which makes them more susceptible to tick vector infestations^41,42^. Additionally, unusual reproductive behaviors of females may cause stress, leading to reduced their immunity and increased tolerance of tick-borne diseases.

The current findings show that the prevalence rate of *B. vogeli* was highest in dogs aged from > 2 to ≤ 4 years. This result was consistent with previous results from Obeta et al.^35,43^. This is most likely due to decreased maternal immunity and resistance in dogs of this age, as well as repeated tick infestations^35,44^. We believe that dogs in this category are active and, if given the opportunity, like to roam randomly, which predisposes them to tick infestations.

The prevalence of *B. vogeli* infection was significantly associated with the presence of ticks. This result is consistent with findings of previous research^33^, which reported that *B. vogeli* was transmitted with *R. sanguineus* s. l. ticks. Therefore, the distribution of ticks among dogs in both urban and rural areas of Egypt would lead to increased susceptibility of dogs to *B. vogeli* infection^45^.

Moreover, the most detectable tick species was observed among examined dogs was *R. sanguineus* s.l. which come in agreement with previous report by Hassanen^23^. As expected, dogs that did not receive adequate care or regular application of suitable acaricides and repellents to reduce the number of ticks they carried were generally associated with a higher prevalence of vector-borne diseases such as babesiosis. These findings are directly in line with those of Araujo et al.^46^, who found a strong correlation between levels of *B. vogeli* infection and lack of veterinary care. The material from which the shelter floor was made was also found to be a significant risk factor in this investigation. Compared with dogs housed in shelters with paved floors, the risk of *B. vogeli* infection was considerably higher in dogs raised in earthen-floor shelters. Paved floors are easier to clean than earth floors and provide less favorable conditions for vector larvae to spread^47^.

Understanding the evolutionary relationships between *B. vogeli* isolates is essential to conduct an in-depth intra-specific diversity analysis that will help to improve prevention and management of the spread of this bacterium. Sequence analysis of partial 18S rRNA gene from an Egyptian isolate obtained in this study showed a high degree of similarity with 18S rRNA partial sequences isolated from different *B. vogeli* isolates infecting dogs from various countries*.* Indeed, a phylogenetic tree was generated and the position of our Egyptian isolate confirmed that the positive dogs were infected with *B. vogeli,* as previously reported by Hassanen^23^ in the same country.

## Conclusion

Overall, epidemiological analysis performed in this study by multivariable logistic regression showed a strong association between the prevalence of *B. vogeli* in dogs and whether or not they were infested with ticks and the type of floor used for their shelters. Phylogenetic analysis confirmed that the partial 18S rRNA sequence identified herein indicated that the revealed isolate of *B. vogeli* were related to those previously infecting Egyptian dogs and other *B. vogeli* worldwide isolates*.*

## Supplementary Information

Supplementary Information.

## Data Availability

All data that were generated or analysed during this study are included in this published article and its additional files.
