# Supplementary material for: Molecular survey and phylogenetic analysis of Babesia vogeli in dogs
Source: Sci Rep. 2022 Apr 28;12:6988. doi: 10.1038/s41598-022-11079-x (PMC9050727; doi:10.1038/s41598-022-11079-x)
Supplement: Supplementary file 1 — Supplementary Information. [file 41598_2022_11079_MOESM1_ESM.pdf]

Benha university
Faculty of Veterinary Medicine
Animal medicine Department

ANIMAL OWNER INFORMED CONSENT FORM FOR USE OF ANIMALS FOR RESEARCH

PART A - INFORMATION SHEET

As the owner or duly authorised agent for the owner, you have been asked to have your animal participate in a research study. Your informed consent is required prior to this use. Please read this document and the Consent Form carefully and feel free to ask any questions you might have.

**Animal Project Title:**	**Molecular survey and phylogenetic analysis of *Babesia vogeli* in dogs**			
**AREC Approval No:**	BUFVTM	**Approval Period Dates**:1/1/2019 to 30/11/2019		
**Principal Investigator Name:**	Abdelfattah Selim			
**School/Department/Centre:**	Animal Medicine			
**Contact Details:**	*Phone:*	Click here to enter text.	*Email:*	Abdelfattah.selim@fvtm.bu.edu.eg
**Co-Investigator(s) Name:**	Click here to enter text.			
**School/Department/Centre:**	Click here to enter text.			
**Contact Details:**	*Phone:*	Click here to enter text.	*Email:*	Click here to enter text.
**The person responsible for the animal(s) during the research study:**	Amir hamed			
**Contact Details:**	*Phone:*	Click here to enter text.	*Email:*	Adelk571@yahoo.com
**The location where the animal(s) participation/research study occurs**	Qalyubia, Kafr ELsheikh, Red Sea			
**Aims and benefits of the Research Study:**	**Babesia vogeli in dogs**			
**Duration of animal(s) participation:**	One year			
**Description of animal(s) procedures to be carried out:**	Collection of blood samples from cephalic vein			
**Possible benefits to the animal(s):**	Determine the prevalence of the disease			

Voluntary Participation:

The participation of your animal is voluntary, and you may withdraw your animal(s) for any reason at any time without a need for justification. If you do not wish to participate, you do not have to provide any reason for your decision. Refusal to participate or withdraw from the research will in no way affect the care to which animal participants are otherwise entitled. If you withdraw from the study before the completion of the study, any data collected about your animal will be retained by FVTM for analysis.

Unforeseen Risks:

Unforeseen risks might arise at any time during the research study. The research investigators will promptly inform owners of all animals participating in the study of any new information that may affect their willingness to participate.

Termination of Participation by Principal Investigator:

The Principal research investigator(s) have the right to terminate the research study for any and/or all participants at any time for any valid reason.

Financial Implications:

There will be no cost to you for the participation of your animal in the research study. You will not be charged for any of the procedures performed solely for the study's purposes. You will receive no reimbursement for the participation of your animal in the research study. All unrelated costs for diagnosis, management, and treatment of your animal are your responsibility (relates to veterinary studies). FVTM does not provide compensation or therapy for any injuries or losses that may occur as a result of participation. If the animal is insured, you are advised to notify the insurer of involvement in the research project, preferably before the participation, and attain a formal consent pertaining to Insurance liabilities.

Knowledge Transfer/Publication of Research Findings:

(Indicate how and to whom the research findings will be reported. Indicate how consenting owners may find out about the results of the research study. This may not apply to all research, such as commercial-in-confidence research). NOTE: For commercial -in -confidence, the owner needs to know this before the research and should be part of the agreement/consent.

Confidentiality:

Owner and animal confidentiality will be maintained. There will be no identification of owners of the animal(s) made when reporting or publishing the data arising from this study.

CONSENT FORM FOR ANIMAL PARTICIPATION IN RESEARCH

PART A - NAME & IDENTIFICATION OF ANIMAL(S)

**Name:**	Click here to enter text.
**Species:**	Dogs
**Breed:**	different
**Sex:**	Male and female
**Age:**	Different ages

PART B - CONSENTING OWNER/AUTHORISED AGENT

Name:	Amir hamed	Proof of ownership shown/ documented:	Click here to enter text.
Signature:	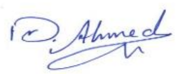	Date:	1/1/2019
Address:	Cairo, Egypt	Telephone/Email:	Adelk571@yahoo.com
